# Official Development Assistance and Private Voluntary Support for Reproductive, Maternal, Neonatal, and Child Health in Guinea-Bissau: Assessing Trends and Effectiveness

**DOI:** 10.3390/children12060717

**Published:** 2025-05-30

**Authors:** Anaxore Casimiro, Joana Branco, Réka Maulide Cane, Michel Jareski Andrade, Luís Varandas, Isabel Craveiro

**Affiliations:** 1 Global Health and Tropical Medicine, GHTM, Associate Laboratory in Translation and Innovation Towards Global Health, LA-REAL, Instituto de Higiene e Medicina Tropical, IHMT, Universidade NOVA de Lisboa, UNL, 1349-008 Lisboa, Portugal; rica.cane@gmail.com (R.M.C.); varandas@ihmt.unl.pt (L.V.); 2 Unidade Local de Saúde de São José, ULS São José, Hospital Dona Estefânia, HDE, 1169-045 Lisboa, Portugal; joana.r.branco@ulssjose.min-saude.pt; 3 NOVA Medical School, Faculdade de Ciências Médicas, Universidade NOVA de Lisboa, Campo Mártires da Pátria 130, 1169-056 Lisboa, Portugal; 4 Instituto Nacional de Saúde, INS, Ministério da Saúde, MISAU, Marracuene 264, Maputo, Mozambique; 5 Nutrition Department, Universidade Estadual do Centro-Oeste, UNICENTRO, Guarapuava 85040-167, Brazil; micheljareski@gmail.com

**Keywords:** reproductive health, maternal health, neonatal health, child health, foreign aid, official development assistance, private voluntary support, Muskoka2 methodology, Guinea-Bissau, West Africa

## Abstract

Background: Reproductive, maternal, neonatal, and child health (RMNCH) remains a key priority for official development assistance and private voluntary assistance (ODA+) in low-income countries. In Guinea-Bissau, maternal and child mortality rates remain high, with the healthcare system heavily dependent on foreign aid. This study analyzes ODA+ trends for RMNCH in Guinea-Bissau from 2002 to 2018 and assesses its impact on maternal, neonatal, infsupplent, and under-five mortality rates. Methods: We used data from the OECD Creditor Reporting System and applied the Muskoka2 methodology to estimate RMNCH-related disbursements. Funding trends were categorized by donor type and RMNCH subsectors. A longitudinal analysis used regression models to assess the relationship between aid categories and mortality outcomes. Results: RMNCH funding accounted for 8.9% of total ODA+ to Guinea-Bissau, with most aid directed toward child health. Models revealed a negative association between child health funding and under-five and infant mortality, while reproductive health funding showed no significant correlation with maternal or neonatal mortality. Conclusions: Although variable, ODA+ for RMNCH in Guinea-Bissau has helped reduce child mortality. However, maternal and neonatal mortality require targeted interventions and improved coordination. Fluctuating aid disbursements emphasize the need for sustainable health financing and stronger donor alignment with national priorities.

## 1. Introduction

Reproductive, maternal, neonatal, and child health (RMNCH) has deserved special attention from the international community over the years, being one of the prioritized areas for foreign aid, especially in low-income countries [1,2,3]. It played a central role in the Millennium Development Goals (MDGs) and remains crucial to the Sustainable Development Goals (SDGs), especially SDG 3.8 - which aims to achieve universal health coverage (UHC), including financial risk protection [4,5,6].

According to the Organization for Economic Cooperation and Development (OECD), foreign aid encompasses financial flows, technical assistance, and commodities aimed at promoting economic development and welfare. It is typically delivered through grants or concessional (subsidized) loans. Foreign aid includes three main categories: Official Development Assistance (ODA), Official Assistance (OA), and Private Voluntary Assistance (PVA), the latter of which involves contributions from non-governmental organizations and private entities [7].

While foreign aid has contributed positively to global development [8,9,10], it has also faced significant criticism, particularly in Africa, where weak institutions and unstable governance have cast doubt on its effectiveness. Some authors argue that aid can foster dependency, fuel corruption, and produce limited long-term results [11,12]. In the health sector, the impact of foreign aid remains debated. Some studies highlight its success in addressing specific health challenges—such as reducing malaria, HIV, and tuberculosis and improving maternal and child mortality rates. Others suggest that foreign aid has little to no effect on broader health outcomes, particularly in reducing child mortality [13,14]. Some aid critics argue that while health aid has caused measurable improvements in health outcomes, the frequency of poor implementation and lack of donor coordination has often led to duplicate efforts and corruption, causing aid funds to disappear, thus suggesting that factors—such as domestic health expenditures, economic growth, and governance quality—are the key drivers of improvements in health outcomes [15,16].

Motivations for foreign aid vary widely, ranging from geopolitical and economic interests to philanthropic and humanitarian goals [8,17,18,19]. Some authors argue that while development-oriented assistance is often channeled through multilateral institutions, bilateral aid is frequently aligned with donor countries’ strategic priorities, favoring countries of geopolitical relevance [20,21].

Guinea-Bissau, a West African nation with a population of 2.15 million [22], ranks among the poorest in the world, with a per capita GDP of $2278.02 (PPP, constant 2021 international dollars) and a Human Development Index (HDI) of 0.483, placing it 179th out of 193 countries [23]. Since gaining independence from Portugal in 1974, Guinea-Bissau has faced persistent political instability, weak governance structures, multiple coups d’état, and the emergence in the last years of transnational drug trafficking networks. These factors have weakened institutional capacity and hindered the consistent delivery of public services, including health care [24,25,26].

As a fragile context, Guinea-Bissau relies heavily on foreign aid to sustain its healthcare system and deliver essential services (Supplementary Figure S1). At the same time, domestic general government health expenditure (GGHE-D) has remained consistently limited over the years. Between 2002 and 2018, domestic general government health expenditure remained consistently low, accounting for 0.4% to 0.6% of the Gross Domestic Product (GDP) [27,28] (Supplementary Figure S2). This has contributed to systemic challenges in healthcare delivery, particularly in reproductive, maternal, neonatal, and child health (RMNCH).

Despite targeted efforts and external support, RMNCH outcomes remain concerning. Guinea-Bissau did not achieve Millennium Development Goals (MDGs) 4 and 5, which aimed to significantly reduce child and maternal mortality by 2015 [27].

The country continues to report high neonatal and under-five mortality rates, compounded by limited access to skilled birth attendants, weak referral systems, and inadequate postnatal care coverage. Similarly, reproductive health services remain underutilized due to barriers to access to family planning, low contraceptive prevalence, and sociocultural constraints [28].

While a small body of research has examined development assistance for health in conflict-affected or fragile settings [29,30], most studies either focus narrowly on reproductive health or assess the link between development assistance for health and disease burden. Few studies have disaggregated aid flows or evaluated their impact across RMNCH subsectors. This limited evidence base leaves critical questions unanswered regarding whether—and how—foreign aid has influenced RMNCH outcomes in unstable settings like Guinea-Bissau. To address this gap, the present study analyzes trends in ODA and private voluntary assistance (ODA+) directed toward RMNCH in Guinea-Bissau from 2002 to 2018 and assesses its association with maternal, neonatal, infant, and under-five mortality.

The choice of Guinea-Bissau as a case study reflects the personal and professional commitment of the first author, a national of Guinea-Bissau with long-standing involvement in maternal and child health in the country. Given Guinea-Bissau’s ongoing political instability, dependence on external aid, and persistent health challenges, it offers a relevant and underexplored context for examining the effectiveness of foreign assistance.

## 2. Materials and Methods

### 2.1. Study Design

We analyzed ODA+ disbursement data from all donors reporting aid disbursements to the Creditor Reporting System (CRS) of the OECD as of 6 May 2021.

### 2.2. Data Gathering

We gathered ODA+ disbursement data from all donors reporting aid disbursements to the OECD CRS. The CRS was established in 1973 by the OECD to collect information on individual aid loans and, later, grants, complementing the recording of aggregate flows of ODA [31]. This dataset included 148 donors (55 bilateral, 65 multilateral, and 28 private donors) covering 2002–2018. Data from the Muskoka2 initiative was also incorporated [32,33]. Muskoka2 consists of an automated algorithm applied to aid data reported to the OECD’s CRS aid activities database, which estimates the monetary value of funding that directly influences RMNCH outcomes globally, by year, by recipient country, and by the donor, rather than only funding earmarked for RMNCH, including funding targeted at specific diseases, such as HIV and malaria, health system strengthening, water and sanitation sector and shares of funding for the humanitarian sector [32].

### 2.3. Data Disbursement Classification

Disbursements were categorized based on donor type in line with the OECD definitions. Specifically, we classified disbursements into three main categories: (1) bilateral aid from individual countries, (2) multilateral aid from international institutions, including Global Health Initiatives, and (3) private voluntary assistance from non-governmental organizations, religious groups, charities, foundations, and private companies. Our analysis focused on disbursements that the OECD identifies as most accurately reflecting actual international transfers of financial resources, goods, or services [34].

### 2.4. Data Estimations and Coding

We applied the Muskoka2 methodology [32,33] to the CRS database to estimate RMNCH disbursements to Guinea-Bissau from 2002 to 2018. The study period spans 2002–2018, chosen for data availability and policy relevance in Guinea-Bissau’s post-conflict recovery.

RMNCH disbursements include activities that improve access for women and children to integrated health interventions, strengthen health systems, and build RMNCH-specific workforce capacity [35]. Expenditures were categorized as observed in Table 1.

Additional funding categories, such as health system strengthening, general healthcare, humanitarian health, and condition-specific funding (e.g., HIV/AIDS, malaria), were proportionally allocated to RMNCH based on CRS purpose codes and refined using data on disease burden, demographics, and government health expenditure [32,38,39]. Percentages (0–100%) were assigned to CRS disbursements by purpose code, with 25 out of 223 codes identified as contributing to RMNCH [32] (Supplementary Tables S1–S3).

In this study, ODA+ for the Health Sector (HS) was defined as the sum of categories I.2 (Health, Total) and I.3 (Population Policies/Programmes & Reproductive Health) from the CRS database [40] and ODA+ to all sectors (AS) represents the total ODA+ to Guinea-Bissau.

### 2.5. Data Categorization and Study Variables

#### 2.5.1. Data Categorization

Annual RMNCH assistance was disaggregated by donor type (bilateral, multilateral, private) and RMNCH category (RH, MNH, CH) for 2002–2018. Disbursement data are presented, unless otherwise specified, in constant 2018 US dollars using the Development Assistance Committee deflators, with data management performed in Microsoft Excel (version 16) [41].

#### 2.5.2. Study Variables

We used four mortality rates as dependent variables to assess the effectiveness of aid: (1) Neonatal mortality rate -deaths per 1000 live births during the first 28 days of life, (2) Infant mortality rate -deaths per 1000 live births of children under one year of age; (3) Under-five mortality rate -deaths per 1000 live births of children under five and (4) Maternal mortality rate - deaths per 100,000 live births due to complications of pregnancy or childbirth [28,42]. These indicators represent specific demographic groups and health outcomes related to maternal and reproductive health, childbirth, and child growth and were sourced from the World Development Indicators Database [28].

Based on current literature, we incorporated several control variables known to influence mortality rates, including Gross Domestic Product (GDP) per capita, life expectancy at birth, sanitation rate, fertility rate, use of modern contraceptives, and vaccination coverage against measles and Diphtheria, Tetanus, and Pertussis (DTP) [22,28,43]. More details on these variables are available in Supplementary Table S4.

**Figure 1:**
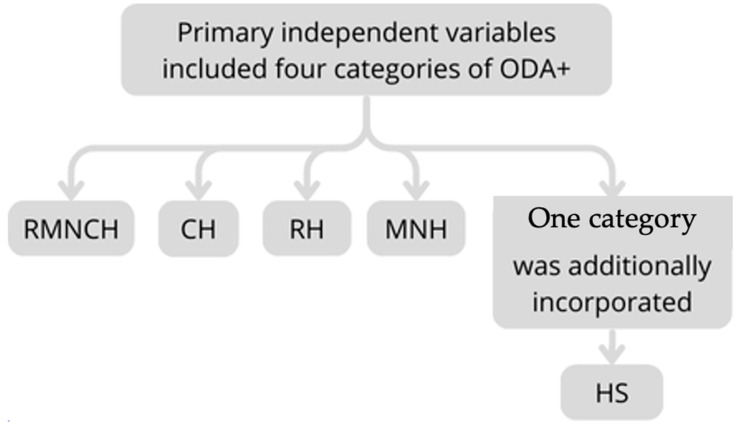
Primary independent variables.

Primary independent variables included four ODA+ categories: RMNCH, CH, RH, and MNH, along with an additional category, HS (Figure 1). This study controlled RMNCH, CH, and MNH variables using GDP per capita, life expectancy, sanitation rate, and vaccination coverage (measles and DTP). Meanwhile, RH was controlled using GDP per capita (PPP), fertility rate, and the prevalence of modern contraceptive use. HS was controlled using GDP per capita, life expectancy, sanitation rate, vaccination coverage (measles and DTP), and fertility rate.

### 2.6. Data Modelling and Analysis

#### 2.6.1. Study Hypothesis

The hypothesis proposed in this study is that Official Development Assistance and private voluntary assistance (ODA+) significantly predict maternal, neonatal, infant, and under-five mortality rates, with an expected negative correlation between aid disbursements and mortality rates.

#### 2.6.2. Model Development

We designed a quasi-experimental longitudinal analysis [44,45], developing fifteen models after excluding control variables exhibiting multicollinearity (Supplementary Table S5).

Each model assessed the relationship between a specific independent variable and a health-related mortality outcome, controlling for relevant covariates (Table 2).

#### 2.6.3. Data Analysis

Multiple regression analysis was performed to assess this relationship, focusing on the β2 coefficient to measure the impact of ODA+ on mortality rates. Several statistical tests were conducted to validate the linear regression assumptions, including a correlation matrix to identify multicollinearity, the Breusch-Pagan/Cook-Weisberg test for homoscedasticity, the Breusch-Godfrey LM test for autocorrelation, and the Shapiro-Wilk W test to assess the distribution type. Multicollinearity was addressed by excluding all control variables except GDP per capita. Quadratic terms were introduced in the eighth, ninth, and eleventh models to address the non-linear effects identified. Homoscedasticity was not observed in any model, and a non-normal distribution was identified in the ninth model. All variables were logarithmically transformed to interpret regression coefficients as percentages. A significance level of α = 0.05 was applied. Data analysis was conducted using Stata Statistical Software (Release 18) [46].

## 3. Results

### 3.1. Flows of ODA+ Funding in Guinea-Bissau

Between 2002 and 2018, Guinea-Bissau received ODA+ disbursements totaling approximately $2.3 billion across various sectors, including social and economic infrastructure, production, commodity aid, and humanitarian assistance. These disbursements represented between 7.6% and 26.7% of the country’s Gross National Income (GNI). During this period, a total of $364 million was allocated to the health sector, encompassing health and population policies as well as reproductive health. This accounted for 16.4% of total ODA+, translating to $6.6 and $23.6 per capita. Of this health sector funding, $197.4 million (8.9% of total ODA+) was directed to Reproductive, Maternal, Neonatal, and Child Health (RMNCH) activities (Figure S3). RMNCH funding experienced a 237% increase, rising from $4.1 million in 2002 to $13.8 million in 2018, significantly outpacing the 101% growth in overall ODA+. The average annual growth rates for total ODA+ and RMNCH-specific funding were 15.8% and 14.8%, respectively. Overall, total ODA+, health sector aid, and RMNCH-specific funding financing patterns varied throughout the study period (Table 3).

### 3.2. Donor Variability and Growth Rates

**Figure 2:**
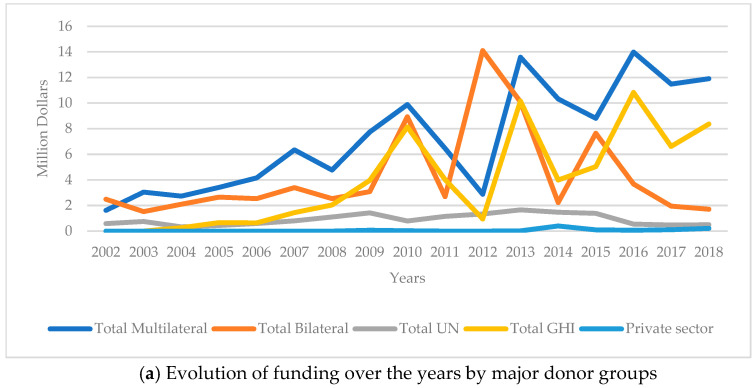

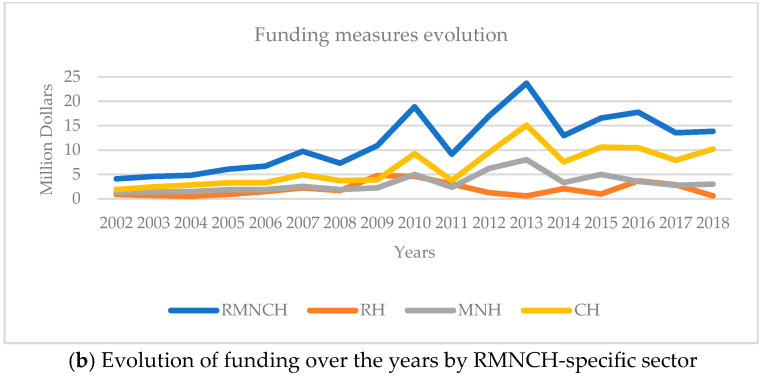
Evolution of funding over the years by major donor groups (**a**) and RMNCH-specific sector (**b**). Abbreviations: UN—United Nations; GHI—Global Health Initiatives; RMNCH—Reproductive, Maternal, Neonatal, and Child Health; CH—Child Health; RH—Reproductive Health; MNH—Maternal and neonatal health.

Funding patterns varied substantially across donor groups rather than consistently over time (Figure 2a,b). The largest bilateral and multilateral donors were the United States of America (USA) (17.4%; $34.4 million), Portugal (11.3%; $22.4 million) and the International Development Association (6%; $11.8 million), while the only private donor was the Bill & Melinda Gates Foundation (0.5%; $0.99 million). Among major donor groups, Global Health Initiatives, particularly the Global Fund, recorded the highest average annual growth rate (102.2% in 2004–2018), followed by bilateral donors, with an annual growth rate of 38.7%.

### 3.3. RMNCH-Specific Funding Allocation

RMNCH-specific funding was primarily directed to child health (56.0%; $110.6 million), followed by maternal and neonatal health (27.3%; $53.9 million) and reproductive health (16.7%; $32.9 million). Multilateral donors provided most of the funding (62.4%; $123.1 million), with bilateral donors contributing 37.1% ($73.3 million) and the private sector 0.5% ($0.99 million). The top donors included the Global Fund ($55.4 million), the USA ($34.4 million), Portugal ($22.4 million), European Union (EU) Institutions ($18.5 million), and the International Development Association ($11.8 million) (Supplementary Figures S3 and S4).

### 3.4. Child Health Funding

Child health support received substantial contributions from key donors, including the Global Fund (25.0% of total child health aid, $27.7 million), the USA (19.5%; $21.6 million), EU Institutions (11.4%; $12.6 million), Portugal (10.7%; $11.8 million), and the Global Alliance for Vaccines and Immunization (GAVI) (10.3%; $11.4 million). Funding primarily targeted three key areas: basic healthcare (21.9%; $24.2 million), which included primary healthcare programs, paramedical and nursing care, and essential medicines and vaccines; malaria control and prevention (20.9; $23.1 million); and basic nutrition (20%; $22.1 million).

### 3.5. Maternal and Neonatal Health Funding

Maternal and neonatal health funding was predominantly supported by the USA (23.0%, $12.4 million), the Global Fund (19.5%, $10.5 million), and Portugal (16.6%, $9.0 million). Focus areas included basic nutrition (25.1%; $13.5 million), malaria control ($10.32 million, 19.1%), and reproductive healthcare ($7.05 million, 13.1%).

### 3.6. Reproductive Health Funding

Reproductive health funding was primarily supported by the Global Fund (52.4%, $17.3 million), Spain (8.5%, $2.8 million), and EU Institutions (6.7%, $2.2 million). Most of the funding was allocated to sexually transmitted diseases (STD) and HIV control (71.0%; $23.4 million), followed by general budget support (8.9%; $2.9 million) and reproductive healthcare (6.2%; $2.1 million). The reproductive healthcare allocation covered initiatives of RH according to OECD [37], such as reproductive health promotion, prenatal and postnatal care (including delivery), infertility prevention and treatment, as well as the prevention and management of abortion-related complications and safe motherhood programs.

### 3.7. ODA+ Funding Allocation

Most ODA+ funding for RMNCH—totaling $192.5 million (97.5%)—was allocated to health and population sectors, water and sanitation, and humanitarian initiatives during this period. Only a small portion, $5 million (2.5%), was designated for general budget support. Among RMNCH support categories, basic nutrition received the largest share at 18.1% ($35.8 million), followed closely by malaria prevention and control at 16.9% ($33.4 million), basic healthcare at 14.5% ($28.7 million), sexually transmitted disease (STD) and HIV control at 12.1% ($23.9 million), and medical services at 9.0% ($17.8 million).

### 3.8. Funding and Mortality Trends

Table 4 and Table 5 illustrate trends in funding measures and mortality rates over the study period. While all mortality rates declined, maternal mortality showed an exception in 2017 (Table 5). The most substantial reductions occurred during the first decade of the study, with annual declines surpassing the average annual reduction (Table 5).

Despite overall growth in aid measures, certain years saw reductions in financial assistance, particularly in the RH aid category. Nevertheless, RH aid exhibited the highest average annual increase among all aid measures, rising by 33.18% per year.

### 3.9. Aid Measures and Mortality Rate Associations

Regression models demonstrated a negative association between aid measures and corresponding mortality rates, except for ODA+ for reproductive health (RH), which showed no correlation with neonatal and maternal mortality. GDP per capita emerged as the most influential control variable, consistently predicting significant reductions in mortality rates across all models. Regarding ODA+ to all sectors, no significant correlation was found with any of the mortality rates (*p* > 0.05). Health-sector-specific ODA+ and GDP per capita explained 88.0–93.0% of the decrease in mortality rates, particularly for infant and under-five mortality (Models 1–4). Similarly, RMNCH funding, combined with GDP per capita, explained 89.0–93.0% of the reductions in mortality rates (Models 5–8).

Models 9–11 demonstrated that funding for Child Health (CH), combined with GDP per capita, explained 94.0% of the reductions in neonatal, infant, and under-five mortality rates. A 1.0% increase in Child Health funding was associated with decreases of 0.16% in infant mortality and 0.17% in under-five mortality. However, the impact on neonatal mortality was less pronounced, showing a less favorable regression coefficient. Although Reproductive Health (RH) funding experienced substantial financial growth, no correlation with neonatal or maternal mortality was observed (Models 12–13) (*p* > 0.05). Models 14–15 showed that Maternal and Neonatal Health (MNH) funding and GDP per capita explained 81.0% and 93.0% of the reductions in neonatal and maternal mortality rates, respectively, with a less favorable regression coefficient for neonatal mortality (Supplementary Table S5).

## 4. Discussion

Our study presents a comprehensive analysis of Official Development Assistance (ODA+) disbursements for Reproductive, Maternal, Neonatal, and Child Health (RMNCH) and their impact on maternal, neonatal, infant, and under-five mortality rates in Guinea-Bissau from 2002 to 2018. Our findings highlight significant financial contributions to RMNCH, with notable increases in child health funding and strong donor participation. However, aid effectiveness varied across categories, with reproductive health (RH) funding showing no significant impact on neonatal and maternal mortality reductions over time. Some authors [47] suggest that higher public spending improves healthcare for pregnant women, thereby reducing maternal mortality rates. Foreign aid might not increase public expenditure in the health sector, such as expanding the healthcare ecosystem and care networks. As a result, financial bottlenecks persist, preventing improvements for these women [47,48].

### 4.1. Trends RMNCH Funding

Between 2002 and 2018, donors prioritized the reproductive, maternal, neonatal, and child health (RMNCH) sectors within the broader context of overall ODA+. The increase in ODA+ funding for RMNCH mirrored the growth in ODA+ across all sectors during this period. This trend reflects global efforts to improve maternal, neonatal, and child health outcomes through concerted donor support and health systems strengthening [49,50,51,52,53]. In the same period under analysis, children aged between 1 month and five years benefited from more than half of the funding for reproductive, maternal, neonatal, and child health due to increased funding from donors to immunization projects and malaria control programs, as observed in other studies [54,55].

### 4.2. RMNCH Funding and Mortality Rates

Our regression models demonstrated a strong negative association between CH funding and infant and under-five mortality rates, suggesting that targeted investments in child health interventions significantly contributed to mortality reduction in Guinea-Bissau. Funding directed at child health demonstrated the strongest associations, explaining 94.0% of reductions in neonatal, infant, and under-five mortality combined with GDP per capita. This highlights the effectiveness of investments in primary healthcare, malaria prevention, and basic nutrition, which accounted for a significant share of child health funding. Similarly, maternal and neonatal health (MHN) funding and GDP per capita explained 81.0% and 93.0% of the reduction in the neonatal and maternal mortality rate, respectively, with a less favorable regression coefficient for neonatal mortality in Guinea-Bissau. In contrast, reproductive health (RH) funding showed the highest annual financial growth with no correlation with neonatal or maternal mortality reductions. This suggests potential inefficiencies in RH aid allocation, limitations in its short-term impact, or that funding levels remain insufficient to address the complex drivers of maternal and neonatal mortality. The lack of correlation may also stem from the nature of RH interventions, which primarily focus on long-term behavioral and systemic changes (such as contraception use and family planning policies) rather than immediate life-saving measures like emergency obstetric care, neonatal resuscitation, or treatment for severe pregnancy complications like eclampsia or hemorrhage [56,57].

### 4.3. Donor Contributions and Funding Variability

Our findings revealed that multilateral donors were the primary contributors to RMNCH funding in Guinea-Bissau, accounting for 62.4%, while bilateral donors also played a significant role, contributing 37.1%. The growing involvement of Global Health Initiatives, including the Global Fund, in Guinea-Bissau since 2004 has influenced the funding trends. Notably, there has been a substantial increase in funding since 2009. Additionally, GAVI has played a significant role in financing projects to improve reproductive, maternal, neonatal, and child health, a trend observed by other researchers [55,58]. Notably, private sector contributions were minimal, highlighting an opportunity to engage more non-state actors in health financing for Guinea-Bissau. Since 2009, private foundations, represented by the Bill and Melinda Gates Foundation, have provided a modest but steady share of RMNCH funding.

This study verified that donors maintained constant support for RMNCH despite the fragile context and periods of significant governance disruptions in Guinea-Bissau between 2002 and 2018 [24,25]. Our study revealed peaks in aid disbursements for RMNCH in 2007, 2010, 2014, and 2016. These years were marked by intense political and military instability in Guinea-Bissau. In 2007, the country witnessed the assassination of a high-ranking military officer and the passage of a no-confidence motion against the government. In 2010, a mutiny within the armed forces led to the arrest of the acting prime minister. The 2014 legislative and presidential elections were followed by institutional conflict between the presidency and the government. In 2016, a parliamentary censure motion against the government further deepened political strife. These surges in disbursement likely reflect the increased reliance on external aid during periods of significant domestic instability, when the state faced substantial constraints in fulfilling its financial responsibilities in the health sector.

Our findings reveal significant annual fluctuations in the disbursement of donor assistance across various areas, a trend observed in similar studies conducted in Kenya, Tanzania, Uganda, and Zambia [54,59]. This can be explained by multiple allocation policies of foreign donors and country-specific characteristics [60,61,62].

Consistent with previous research [32], we observed that RMNCH funding was highly concentrated among a few donors, notably the Global Fund, USA, Portugal, EU Institutions, International Development Association, and GAVI. We also observed that the priority of funding was given to basic nutrition, malaria prevention and control, and basic health before the control of sexually transmitted diseases, including HIV, which was reported as a priority area in other studies [63]. While significant, maternal and neonatal health funding appears to have less impact on neonatal mortality in Guinea-Bissau, suggesting that targeted interventions in this area require further strengthening. Investments in maternal nutrition, skilled birth attendance, and postnatal care should be prioritized to address persistent gaps.

Similarly to previous studies [63,64], the overwhelming focus of reproductive health funding on sexually transmitted diseases and HIV control (71.0% of total reproductive health aid) may have overshadowed other critical areas, such as safe motherhood initiatives and access to family planning services. Thus, balancing investments across these areas could enhance the overall impact of reproductive health aid. Almost all ODA+ to RMNCH in this period was channeled as projects rather than general budget support, as observed in other studies [54,55], which displays a pattern of health financing that could raise questions about national ownership and long-term sustainability. The question of ownership was raised in the Paris Declaration, reinforced by the Accra Agenda in 2008 [65] and the Busan Partnership for Effective Development Cooperation in 2011, in which donors and partners were encouraged to adhere to the principles of national ownership of development strategies, alignment of aid with national priorities, harmonization of donor activities, focus on results, mutual accountability, predictability, and transparency [66]. This issue was reinforced by UHC 2030, which resulted from the transformation of the International Health Partnership + (IHP +), whose role in cooperation and national ownership was reaffirmed in the Political declaration of the high-level meeting on universal health coverage in 2019 [67]. Based on our data, considering the annual variations in funding and its distribution across different areas, it is unclear whether there is a real effort to harmonize priorities between the multiple donors and the national authorities of Guinea-Bissau. This fact also suggests the need to explore and develop new strategies and actions that can contribute to this harmonization by engaging all relevant stakeholders involved in foreign aid in a participatory and collaborative process.

### 4.4. Influence of Socioeconomic Factors on Mortality Reduction

Beyond ODA+ disbursements, our findings highlight the crucial role of economic and social determinants in shaping health outcomes in Guinea-Bissau. Among these, GDP per capita emerged as the most significant predictor of mortality reduction across all models, underscoring the broader impact of economic growth on health improvements. Additionally, variables such as sanitation access, fertility rates, and vaccination coverage played crucial roles in influencing maternal and child health outcomes. Cross-country studies have shown that expanded access to sanitation significantly reduces neonatal and under-five mortality [68,69,70]. At the same time, vaccination programs play a critical role in lowering the burden of infectious diseases and enhancing child survival [71,72]. These findings underscore the importance of integrated, multisectoral strategies for advancing maternal and child health [73,74]. While foreign aid remains essential, it should be complemented by broader socioeconomic development policies and a robust framework addressing the social determinants of health—particularly the structural factors—to ensure sustainable improvements.

### 4.5. Strengths and Limitations

To our knowledge, this is the first study to examine comprehensively the official development assistance and private voluntary assistance (ODA+) trends for RMNCH in Guinea-Bissau and evaluate their impact on maternal, neonatal, infant, and under-five mortality rates.

While this study offers important insights, it is not without limitations. First, we used data from the CRS database that does not capture all aid flows, namely from donors such as China, Brazil, or India. These countries have supported Guinea-Bissau in the health sector over the years, and it would be interesting to analyze their contribution to RMNCH over the years under study. However, as these countries do not report to the OECD Development Assistance Committee, their assistance was not considered for this work. Additionally, because CRS purpose code allocations rely on donor-provided descriptions, some inconsistencies in project classification may exist over time. Finally, our study reflects a macro-level analysis over a limited time frame and does not explore subnational project details. Despite these considerations, the findings provide an important foundation for understanding RMNCH-related aid in Guinea-Bissau and highlight the need for more granular and comparative analyses in future studies.

## 5. Conclusions

This study highlights the significant progress achieved through RMNCH funding in Guinea-Bissau, particularly in reducing child mortality. However, persistent gaps in maternal and neonatal health outcomes call for targeted strategies to address these challenges. By aligning donor priorities with national health needs and integrating economic growth strategies, Guinea-Bissau can continue to build on these gains and advance towards achieving SDG health targets.

Strengthening financial sustainability by promoting domestic resource mobilization can mitigate fluctuations in donor contributions and enhance program stability.

Ensuring the efficient allocation of RH funding is crucial, as its lack of correlation with mortality reductions suggests a need to reassess intervention strategies.

Policymakers should prioritize scaling up successful interventions in child health and strengthening maternal and neonatal health programs. Greater engagement with the private sector and innovative financing mechanisms could diversify funding sources and enhance sustainability. Finally, ensuring that aid aligns with national health priorities and is effectively implemented is crucial to maximizing its impact. Integrating RMNCH investments with broader health system strengthening efforts will be key to achieving lasting improvements, particularly in maternal and neonatal health outcomes.

Future research should explore qualitative dimensions of aid effectiveness, including governance, healthcare infrastructure, and community engagement in RMNCH programs, while also investigating the impact of donor coordination and alignment with national health priorities to provide further insights into optimizing development assistance for maternal and child health.

## Figures and Tables

**Table 1:** Categorization of Expenditure.

Category	Description
Reproductive Health (RH)	Activities focused on reproductive and sexual health for non-pregnant women, including family planning and population policies [32,36].
Maternal and Neonatal Health (MNH)	Interventions focused on the health of pregnant women and their newborns during pregnancy, childbirth, and the one-month postnatal period [32,37].
Child Health (CH)	Activities aimed at improving children’s health from one month to five years [32,37].

**Table 2:** Regression Model Specifications.

Model	Dependent Variable	Independent Variable	Control Variables
1	Neonatal mortality	HS	GDP per capita, DTP and measles coverage, life expectancy, sanitation, fertility rate
2	Infant mortality	HS	GDP per capita, DTP and measles coverage, life expectancy, sanitation
3	Under-5 mortality	HS	Same as Model 2
4	Maternal mortality	HS	GDP per capita, fertility rate
5	Neonatal mortality	RMNCH	Same as Model 1
6	Infant mortality	RMNCH	Same as Model 2
7	Under-5 mortality	RMNCH	Same as Model 2
8	Maternal mortality	RMNCH	Same as Model 4
9	Neonatal mortality	CH	Same as Model 1
10	Infant mortality	CH	Same as Model 2
11	Under-5 mortality	CH	Same as Model 2
12	Neonatal mortality	RH	Same as Model 1
13	Maternal mortality	RH	Same as Model 4
14	Neonatal mortality	MNH	Same as Model 1
15	Maternal mortality	MNH	Same as Model 4

**Table 3:** Descriptive statistics of the sample for the period 2002–2018.

Dependent Variables	Min.	Mean	Median	Máx.	SD	Annual Average Trend	N
Neonatal mortality	36.70	45.08	45.10	53.20	5.58	↓ 2.29%	17
Infant mortality	54.60	74.48	72.60	99.50	14.72	↓ 3.68%	17
Under-five mortality	82.40	118.01	114.40	163.40	26.50	↓ 4.19%	17
Maternal mortality	648.00	830.59	795.00	1136.00	145.23	↓ 3.38%	17
Funding (US $, Millions, 2018)							
ODA+ all sectors	77.27	130.74	113.07	273.83	50.56	↑15.84%	17
ODA+ Health	8.71	21.43	19.89	40.15	31.44	↑ 13.83%	17
ODA+ RMNCH	4.10	11.61	10.89	23.69	5.78	↑ 14.83%	17
ODA+ CH	1.90	6.51	4.94	15.10	3.84	↑ 23.46%	17
ODA+ RH	0.53	1.94	1.50	4.77	1.40	↑ 33.18%	17
ODA+ MNH	1.34	3.17	2.53	8.03	1.87	↑ 17.24%	17
Control variables							
GDP per capita	1600.65	1703.08	1690.09	1872.31	84.58	↑ 0.83	17
Average life expectancy at birth	50.98	55.98	56.23	60.50	3.19	↑ 1.08	17
Basic sanitation rate (%)	3.01	7.88	7.80	3.20	13.03	↑ 11.11	17
Fertility rate	4.26	5.61	5.06	5.06	0.42	↓1.70	17
Modern contraception (%)	0.059	0.098	0.094	0.154	0.031	↑ 6.20	17
Measles vaccination (%)	66.00	76.00	76.00	83.00	4.78	↑ 0.78	17
DTP vaccination coverage (%)	57.00	77.18	80.00	87.00	9.89	↑ 2.36	17

Abbreviations: DTP—diphtheria, tetanus and polio; SD—standard deviation; ODA+—Official Development Assistance and Private Voluntary Assistance; RMNCH—Reproductive, Maternal, Neonatal, and Child Health; CH—Child Health; RH—Reproductive Health; MNH—Maternal and neonatal health; GDP per capita—gross domestic product per capita (PPP (constant 2017 international $)); Neonatal mortality: deaths per 1000 live births during the first 28 days of life; Under-five mortality: deaths per 1000 live births of children under five; Infant Mortality: deaths per 1000 live births of children under one year of age; Maternal mortality: number of maternal deaths per 100,000 live births due to complications of pregnancy or childbirth; Fertility rate, total: births per woman. Sources: [28,32,33,40].

**Table 4:** Trends in funding measures 2002–2018.

Year	RMNCH	AAGR (%)	RH	AAGR (%)	CH	AAGR (%)	MNH	AAGR (%)
2002	4.1		0.9		1.9		1.3	
2003	4.6	11.4	0.7	22.8	2.5	29.9	1.4	7.2
2004	4.8	5.7	0.5	21.2	2.9	15.5	1.5	1.3
2005	6.1	25.6	0.9	73.9	3.3	15.4	1.9	28.0
2006	6.7	10.3	1.5	63.8	3.3	0.7	1.9	0.8
2007	9.7	45.6	2.3	50.8	5.0	49.5	2.5	34.5
2008	7.3	24.9	1.7	25.2	3.7	24.7	1.9	25.0
2009	10.9	48.9	4.8	181.7	3.9	4.4	2.2	17.7
2010	18.9	73.2	4.6	3.1	9.3	137.9	5.0	123.5
2011	9.2	51.5	3.0	34.4	3.8	59.4	2.4	52.6
2012	17.0	85.5	1.3	58.5	9.5	153.8	6.2	161.5
2013	23.7	39.6	0.6	54.7	15.1	58.7	8.0	29.3
2014	13.0	45.4	2.1	264.0	7.6	49.9	3.3	58.7
2015	16.6	27.8	1.0	51.7	10.6	39.8	5.0	50.1
2016	17.7	7.1	3.7	268.7	10.5	0.9	3.6	28.5
2017	13.5	23.6	2.9	21.4	7.9	24.8	2.8	22.5
2018	13.8	2.0	0.6	79.0	10.2	29.5	3.0	9.0

Abbreviations**:** AAGR—Average annual growth rate; AARR—Average Annual Rate of Reduction; RMNCH—Reproductive, Maternal, Neonatal, and Child Health; CH—Child Health; RH—Reproductive Health; MNH—Maternal and neonatal health.

**Table 5:** Trends in mortality rate 2002–2018.

Year	Infant Mortality	AARR (%)	Under-Five Mortality	AARR (%)	Neonatal Mortality	AARR (%)	Maternal Mortality	AARR (%)
2002	99.5		163.4		53.2		1136	
2003	96.4	3.1	157.6	3.6	52.4	1.5	1100	3.2
2004	93.0	3.5	151.5	3.9	51.7	1.3	1008	8.4
2005	89.6	3.7	145.2	4.2	50.9	1.6	977	3.1
2006	86.2	3.8	138.9	4.3	50.0	1.8	875	10.4
2007	82.7	4.1	132.6	4.5	49.0	2.0	845	3.4
2008	79.2	4.2	126.3	4.8	47.8	2.5	841	0.5
2009	75.8	4.3	120.1	4.9	46.5	2.7	804	4.4
2010	72.6	4.2	114.4	4.8	45.1	3.0	795	1.1
2011	69.6	4.1	109.0	4.7	43.8	2.9	767	3.5
2012	66.7	4.2	103.9	4.7	42.5	3.0	758	1.2
2013	64.2	3.8	99.4	4.3	41.4	2.6	742	2.1
2014	61.9	3.6	95.5	3.9	40.2	2.9	733	1.2
2015	59.9	3.2	91.8	3.9	39.2	2.5	713	2.7
2016	58.0	3.2	88.6	3.5	38.4	2.0	673	5.6
2017	56.3	2.9	85.5	3.5	37.5	2.3	705	4.8
2018	54.6	3.0	82.4	3.6	36.7	2.1	648	8.1

Abbreviations: AAGR—Average annual growth rate; AARR—Average Annual Rate of Reduction; Neonatal mortality: deaths per 1000 live births during the first 28 days of life; Under-five mortality: deaths per 1000 live births of children under five; Infant Mortality: deaths per 1000 live births of children under one year of age; Maternal mortality: number of maternal deaths per 100,000 live births due to complications of pregnancy or childbirth.

## Data Availability

Data generated during this study are available in the article and its Supplementary Information File.

## Supplementary Materials

The following supporting information can be downloaded at: https://www.mdpi.com/article/10.3390/children12060717/s1, Figure S1: External health expenditure (% of current health expenditure) (The World Bank, 2024 [28]); Figure S2: Domestic General Government Health Expenditure (GGHE-D) as % Gross Domestic Product (GDP) (The World Bank, 2024 [28]); Figure S3: RMNCH as a % of all sectors and health and population aid; Figure S4: Total RMNCH financing in Guinea-Bissau by main donors, 2002–2018, in constant 2018 million US dollar; Table S1: Selected key indicators of Guinea-Bissau and West and Central Africa (adapted from The World Bank, 2024 [28]); Table S2: Flow diagram of the study method for RMNCH (adapted from Dingle et al., 2020 [32]); Table S3: Flow diagram of RH, MNH, and CH disbursement attribution by donor and purpose code (adapted from Dingle et al., 2020 [32]); Table S4: Description of variables; Table S5: Trends in mortality rates and funding measures 2002–2018.

## Abbreviations

AS, All Sector Aid; CH, Child Health; DAC, Development Assistance Committee; DAH, Development Assistance for Health; GNI, gross domestic product; HS, Health Sector Aid; MDG, Millennium Development Goals; MNH, Maternal and neonatal health; ODA, Official Development Assistance; OECD, Organization for Economic Cooperation and Development; RMNCH, Reproductive, maternal, neonatal, and child health; RH, Reproductive Health; SDG, Sustainable Development Goals; USD, US dollars.
