# Supplementary material for: Official Development Assistance and Private Voluntary Support for Reproductive, Maternal, Neonatal, and Child Health in Guinea-Bissau: Assessing Trends and Effectiveness
Source: Children (Basel). 2025 May 30;12(6):717. doi: 10.3390/children12060717 (PMC12191337; doi:10.3390/children12060717)
Supplement: Supplementary file 1 [file children-12-00717-s001.zip › children-3630961-supplementary.pdf]

Supplementary Materials

Table S1. Selected key indicators of Guinea-Bissau and West and Central Africa (adapted from The World Bank, 2024)

Key Indicators	Guinea-Bissau (2021)	Western and Central Africa (2021)
**Population, Total**	2.060.721	478.185.907
**Gross Domestic Product per capita, PPP** (constant 2021 international $)	2278.02	4756.31
**Life expectancy at birth, total** (years)	59.7	57.0
**Mortality rate under 5** (per 1,000 live births)	74.5	94.4
**Maternal mortality ratio** (modeled estimate per 100,000 live births) (2020)	725	733
**Neonatal mortality rate** (per 1,000 live births)	34.3	31.3
**Current health expenditure as % of GDP**	8.2	4.1
**Current health expenditure per capita, PPP** (current international $)	174.1	183.9
**Domestic general government health expenditure** (% of GDP)	1.1	0.9
**Domestic general government health expenditure** (% of current health expenditure)	13.8	22.8
**Out-of-pocket expenditure** (% of current health expenditure)	60.0	59.0
**External health expenditure** (% of current health expenditure)	23.2	11.9
**External health expenditure per capita, PPP** (current international $)	40.5	22.0

Abbreviations: GDP, gross domestic product; USD, US dollars; PPP, Purchasing power parity

Table S2. Flow diagram of the study method for RMNCH (adapted from Dingle et al., 2020)

OECD CRS disbursement data			
Does the donor have an RMNCH-specific mandate?			
Yes		*No*	
Institution and RMNCH%		*Purpose Code and RMNCH%*	
GAVI	*91%*	*Health and population sectors (120/130)*	
UNFPA	*49%*	*Basic nutrition*	*100%*

UNICEF	*15%*	*Reproductive health care*	*100%*
		Family planning	*100%*
		Personnel development for population and reproductive health	*100%*
		Health policy and administrative management	*40%*
		Medical education/training	*40%*
		Medical services	*40%*
		Basic health care	*40%*
		Basic health infrastructure	*40%*
		Infectious disease control	*40%*
		Health education	*40%*
		Health personnel development	*40%*
		Population policy and administrative management	*40%*
		Malaria	*Varies**
		Controlling STDs including HIV/AIDS	*Varies**
		Tuberculosis	*Varies**
		Medical research	*0%*
		Water and sanitation sector (140)	
		Basic drinking water supply and basic sanitation	*15%*
		Basic drinking water supply	*15%*
		Basic sanitation	*15%*
		All Other purpose codes in sector	*0%*
		Humanitarian sector (720, 730, 740)	
		Material relief assistance and services	*4,4%*
		Relief co-ordination; protection and support services	*2,1%*
		Emergency food aid	*1,9%*

Disaster prevention and preparedness	*1,5%*
Reconstruction relief and rehabilitation	*1,4%*
Other sectors	
General budget support	*Varies**
All Other purpose codes	*0%*

**Note: The proportion of funding in the malaria, HIV, tuberculosis, and general budget support purpose codes that is considered to support RMNCH varies by year based on publicly available data on either disease burden or government expenditure on health.*

% applied to value to produce RMNCH estimates for each disbursement

RMNCH estimates analyzed to produce Guinea-Bissau estimates of aid for RMNCH by year

CH=child health; CRS=Creditor Reporting System; MNH=maternal and newborn health; GAVI= The Global Alliance for Vaccines and Immunizations; OECD=Organisation for Economic Cooperation and Development; RH=reproductive health; RMNCH=reproductive, maternal, newborn, and child health; STD=sexually transmitted disease; UNFPA=United Nation Population Fund; UNICEF= United Nations Children's Fund

Table S3. Flow diagram of RH, MNH, and CH disbursement attribution by donor and purpose code (adapted from Dingle et al., 2020)

OECD CRS disbursement							
Does the donor have an RMNCH-specific mandate?							
Yes				*No*			
Institution	*RH%*	*MNH%*	*CH%*	*Purpose code*	*RH%*	*MNH%*	*CH%*
GAVI	*0,1*	*1,9*	*89*	*Basic nutrition*	*0,5*	*37,9*	*61,6*
UNFPA	*11,8*	*37,1*	*0,1*	*Reproductive health care*	*15,8*	*58,9*	*25,3*
UNICEF	*0,9*	*3,3*	*10,8*	*Family planning*	*97,3*	*2*	*0,7*
				Personnel development for population and reproductive health	*14,5*	*70,1*	*15,4*
				Health policy and administrative management	*1,9*	*13,5*	*24,6*
				Medical education/training	*1*	*15,1*	*23,9*
				Medical services	*1,8*	*15,7*	*22,5*
				Basic health care	*0,6*	*9,4*	*30*
				Basic health infrastructure	*0,7*	*12,9*	*26,4*

Infectious disease control	0,5	1,5	38
Health education	6,2	11	22,8
Health personnel development	0,6	16,4	23
Population policy and administrative management	23,4	12	4,6
Malaria	0	15	Varies*
Controlling STDs including HIV/AIDS	Varies*	0	Varies*
Tuberculosis	0	0	Varies*
Medical research	0	0	0
Water and sanitation sector (140)			
Basic drinking water supply and basic sanitation	0	0	15
Basic drinking water supply	0	0	15
Basic sanitation	0	0	15
All Other purpose codes in sector	0	0	0
Humanitarian sector (720, 730, 740)			
Material relief assistance and services	0,1	0,9	3,4
Relief co-ordination; protection and support services	0,1	0,5	1,5
Emergency food aid	0	0,6	1,3

Disaster prevention and preparedness	*0*	*0,4*	*1,1*
Reconstruction relief and rehabilitation	*0*	*0,4*	*1*
Other sectors			
General budget support	*Varies**	*0*	*Varies**
All Other purpose codes	*0*	*0*	*0*

**Note: The proportion of funding in the malaria, HIV, tuberculosis, and general budget support purpose codes that is considered to support RMNCH varies by year based on publicly available data on either disease burden or government expenditure on health.*

% applied to value to produce RMNCH estimates for each disbursement

RMNCH estimates analyzed to produce Guinea-Bissau estimates of aid for RMNCH by year

CH=child health; CRS=Creditor Reporting System; MNH=maternal and newborn health; GAVI= The Global Alliance for Vaccines and Immunizations; OECD=Organisation for Economic Cooperation and Development; RH=reproductive health; RMNCH=reproductive, maternal, newborn, and child health; STD=sexually transmitted disease; UNFPA=United Nation Population Fund; UNICEF= United Nations Children's Fund.

Table S4. Description of variables

	Variables description
Gross Domestic Product (GDP) per capita	*represents a country or region's average economic output per person. Our work presents the GDP per capita figure in the 2017 constant PPS. GDP negatively correlates with maternal and under-five mortality rates (Bishai et al., 2016; DerSarkissian et al., 2013; Zhang et al., 2023).*
Life expectancy at birth	*refers to the expected number of years a newborn is projected to live. The relationship between life expectancy, maternal mortality, and under-five mortality rates is influenced by a complex interplay of maternal health, socioeconomic factors, and environmental conditions (Hertz et al., 1994).*
Sanitation rate	*represents the percentage of the population with access to adequate and safe sanitation services. Sanitation is a cornerstone of public health, playing a vital role in reducing maternal and under-five mortality. Access to clean drinking water and basic sanitation minimizes the incidence of multiple pathologies and, thereby, improves the health outcomes of mothers and children (Aziz et al., 2021; Cheng et al., 2012).*
Fertility rate	*represents the average number of children a hypothetical cohort of women would bear by the end of their reproductive years (ages 15–49), expressed as the number of children per woman. The inverse relationship between maternal mortality and fertility rates is well-documented in the literature, with evidence showing that declining fertility has contributed to reductions in maternal mortality in many countries (Jain, 2011; Khudri, 2016). Moreover, high-risk fertility behaviors—such as early maternal age and short birth intervals—are recognized as significant determinants of under-five mortality (Khan et al., 2021).*

Modern contraceptives *refers to the percentage of married or cohabiting women aged 15–49 who currently use, or whose partners use, at least one modern method of birth control. Modern contraception is pivotal in reducing maternal mortality, with studies indicating that increased contraceptive use in developed countries has resulted in a 40% reduction in maternal deaths by preventing unintended pregnancies (Ahmed et al., 2012; Cleland et al., 2012; Stover & Ross, 2010). Furthermore, modern contraceptive methods contribute to lowering under-five mortality rates by reducing high-risk pregnancies and improving child survival outcomes, particularly by decreasing the risks of prematurity and low birth weight (Chikandiwa et al., 2018; Cleland et al., 2012).*

Vaccination coverage against measles *Measles vaccination coverage refers to the percentage of children aged 12–23 months who have received at least one dose of the measles vaccine. It has been a critical tool in reducing under-five mortality globally, not only by directly lowering measles-related deaths (Goldhaber-Fiebert et al., 2010) but also through its non-specific effects, which help reduce mortality from other diseases (Aaby et al., 2010, 2015).*

Diphtheria, Tetanus, and Pertussis (DTP) *represents the percentage of children aged 12–23 months who have received the third dose of the combined DTP vaccine. The DTP vaccine is a cornerstone of childhood immunization programs worldwide and has been extensively studied for its impact on maternal and under-five mortality rates. While some studies have associated DTP vaccination with improved child survival (Breiman et al., 2004), others have raised concerns about its potential link to increased mortality, particularly among girls (Aaby et al., 2012; Higgins et al., 2016).*

Table S5. Trends in mortality rates and funding measures 2002-2018

		Newborn mortality (logged)	***Infant mortality (logged)***	***Under-five mortality (logged)***	***Maternal mortality (logged)***
Aid measures (logged)					
		1st model	*2nd model*	*3rd model*	*4th model*
HS	*coefficient*	-0,113	-0,202	-0,228	-0,206
	P value	0,001	<0.001	<0.001	0,001
	R^2	0,921	0,926	0,927	0,875
		5th model	*6th model*	*7th model*	*8th model*
RMNCH	*coefficient*	-0.091	-0.167	-0.190	-0.176
	P value	0.002**	0.000***	0.000***	0.001**
	R^2	0.917	0.930	0.93	0.890
		9th model	*10th model*	*11th model*	
CH	*coefficient*	-0.091	-0.160	-0.170	
	P value	0.000	0.000	0.000	
	R^2	0.940	0.940	0.940	
		12th model			*13th model*
RH	*coefficient*	-0.006			-0.034
	P value	0.647			0.174
	R^2	0.832			0.730
		14th model			*15th model*

MNH	*coefficient*	*-0.074*			*-0.688*
	P value	*0.002*			*<0.001*
	R^2	*0.806*			*0.927*
Control (logged)					
GDP per capita (coefficient)		*-2,333*	*-2.392*	*-2.738*	*-2.858*

**p<0.05, **p<0.01, ***p<0.001*

Abbreviations: *HS- Health-sector-specific ODA+; RMNCH – Reproductive, Maternal, Neonatal, and Child Health; CH - Child Health; RH - Reproductive Health; MNH - Maternal and neonatal health; GDP per capita – gross domestic product per capita (PPP (constant 2017 international $)*

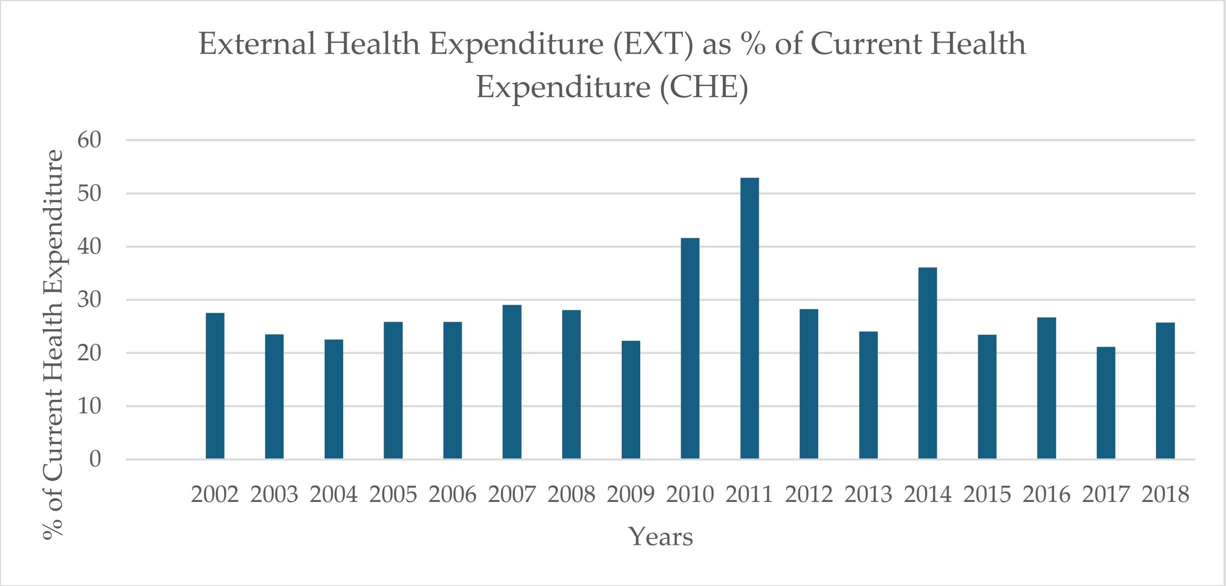

Figure S1: External health expenditure (% of current health expenditure) (The World Bank, 2024)

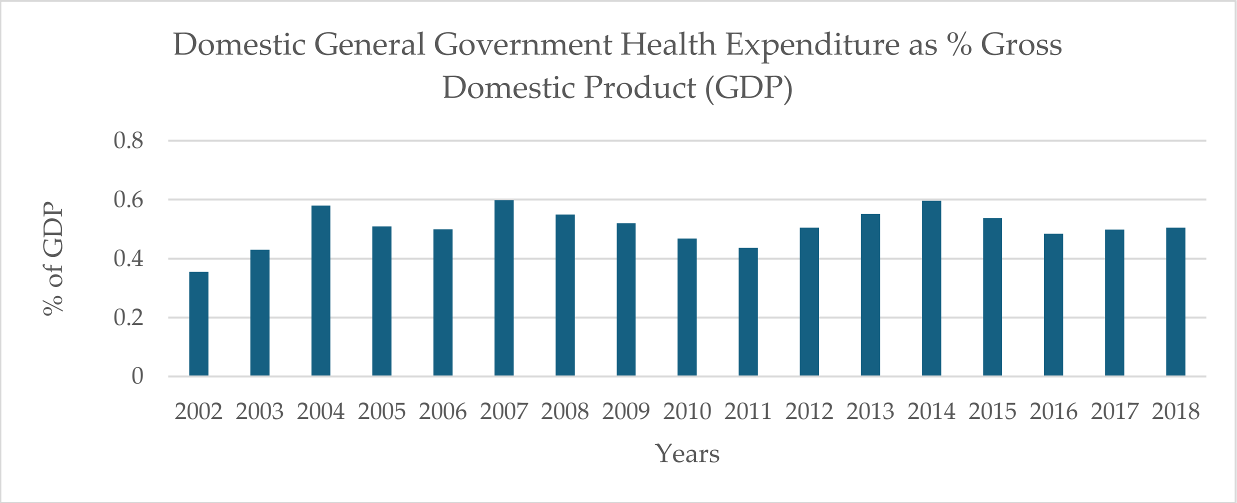

Figure S2: Domestic General Government Health Expenditure (GGHE-D) as % Gross Domestic Product (GDP) (The World Bank, 2024)

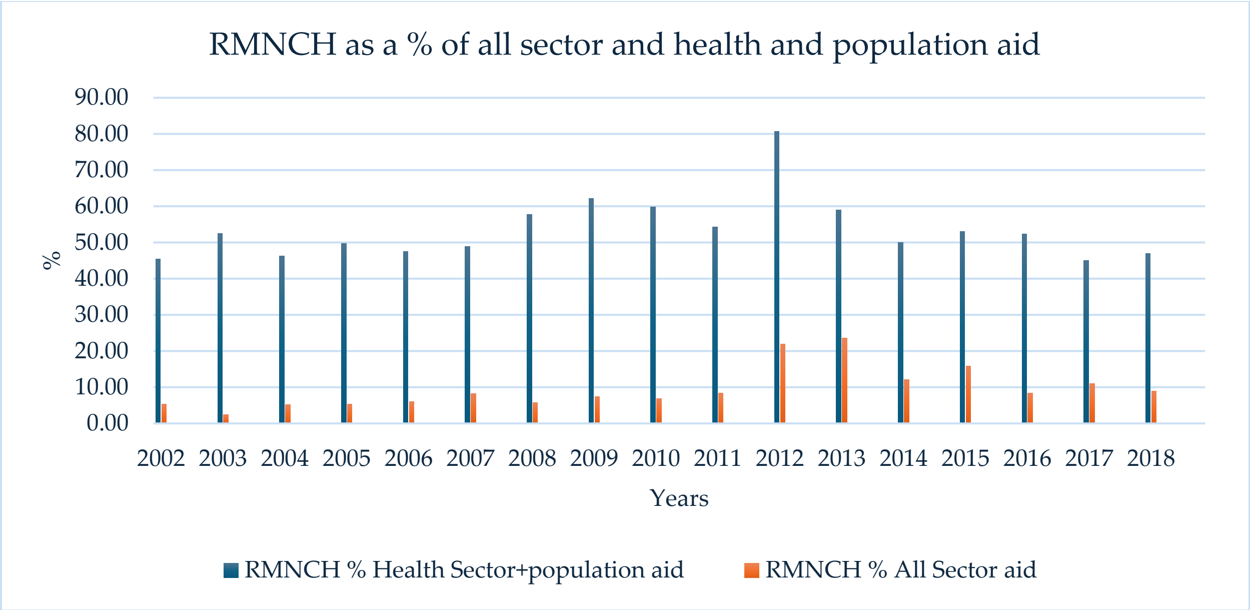

Figure S3: RMNCH as a % of all sectors and health and population aid

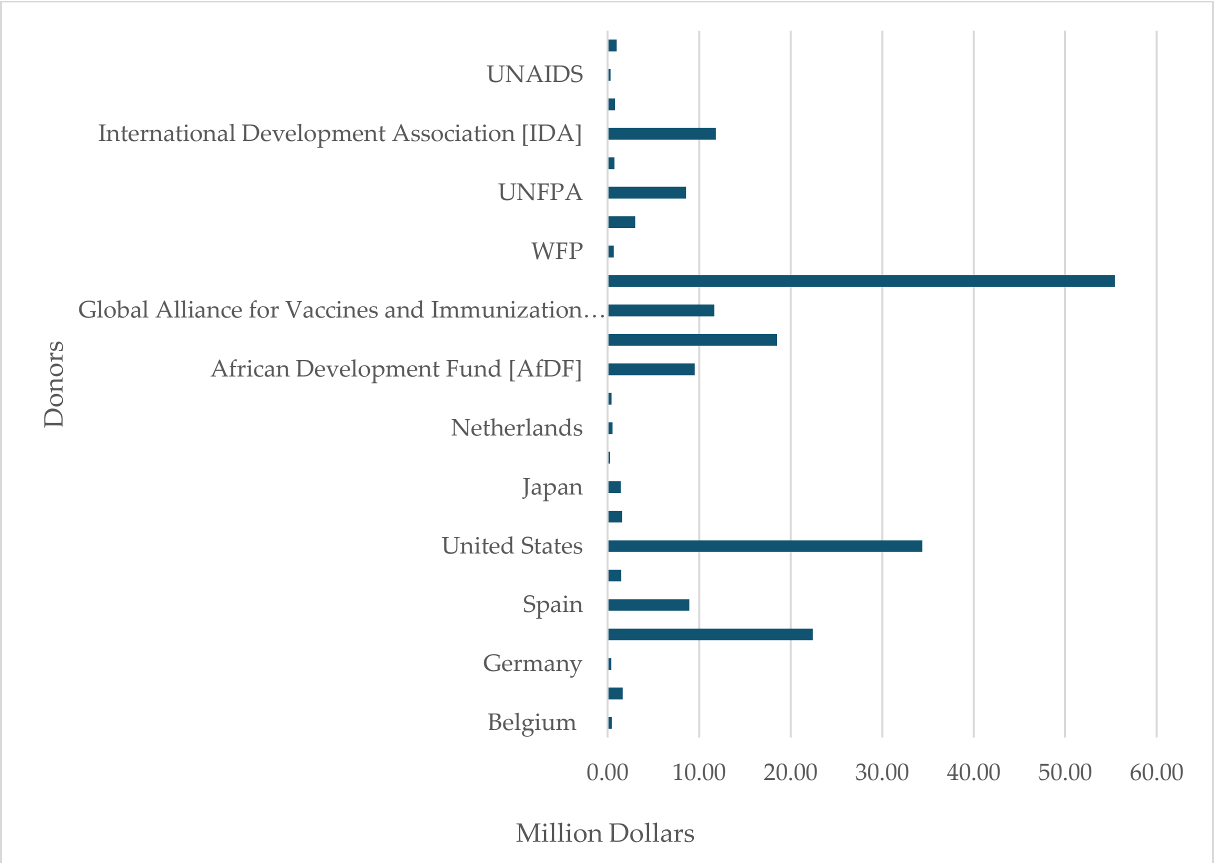

Figure S4: Total RMNCH financing in Guinea-Bissau by main donors, 2002-2018, in constant 2018 million US dollar
Abbreviations: *UNAIDS- Joint United Nations Programme on HIV/AIDS; IMF- International Monetary Fund; UNFPA- United Nations Population Fund; WFP- World Food Programme; UNICEF- The United Nations International Children's Emergency Fund*
